# Supplementary material for: Time-resolved β-lactam cleavage by L1 metallo-β-lactamase
Source: Nat Commun. 2022 Nov 30;13:7379. doi: 10.1038/s41467-022-35029-3 (PMC9712583; doi:10.1038/s41467-022-35029-3)
Supplement: Supplementary file 1 — Supplementary Information [file 41467_2022_35029_MOESM1_ESM.pdf]

Supplementary Information

Time-Resolved β-lactam Cleavage by L1 Metallo-β-Lactamase

Wilamowski, M.[1,2,3], Sherrell, D.A.[4], Kim, Y.[1,4], Lavens, A.[4], Henning, R.W.[5], Lazarski, K.[4], Shigemoto, A.[6], Endres, M.[1], Maltseva, N.[1], Babnigg, G.[1], Burdette, S.C.[6], Srajer, V.[5], Joachimiak, A.[1,2,4*]

[1]Center for Structural Genomics of Infectious Diseases, Consortium for Advanced Science and Engineering, University of Chicago, Chicago, IL 60667, USA; [2]Department of Biochemistry and Molecular Biology, University of Chicago, Chicago, IL 60637, USA; [3]Department of General Biochemistry, Faculty of Biochemistry, Biophysics and Biotechnology of Jagiellonian University, Krakow, 30387, Poland; [4]Structural Biology Center, X-ray Science Division, Argonne National Laboratory, Argonne, IL 60439, USA, [5]Center for Advanced Radiation Sources, University of Chicago, Chicago, IL 60637, USA. [6]Department of Chemistry and Biochemistry, Worcester Polytechnic Institute, Worcester, MA 01609, USA

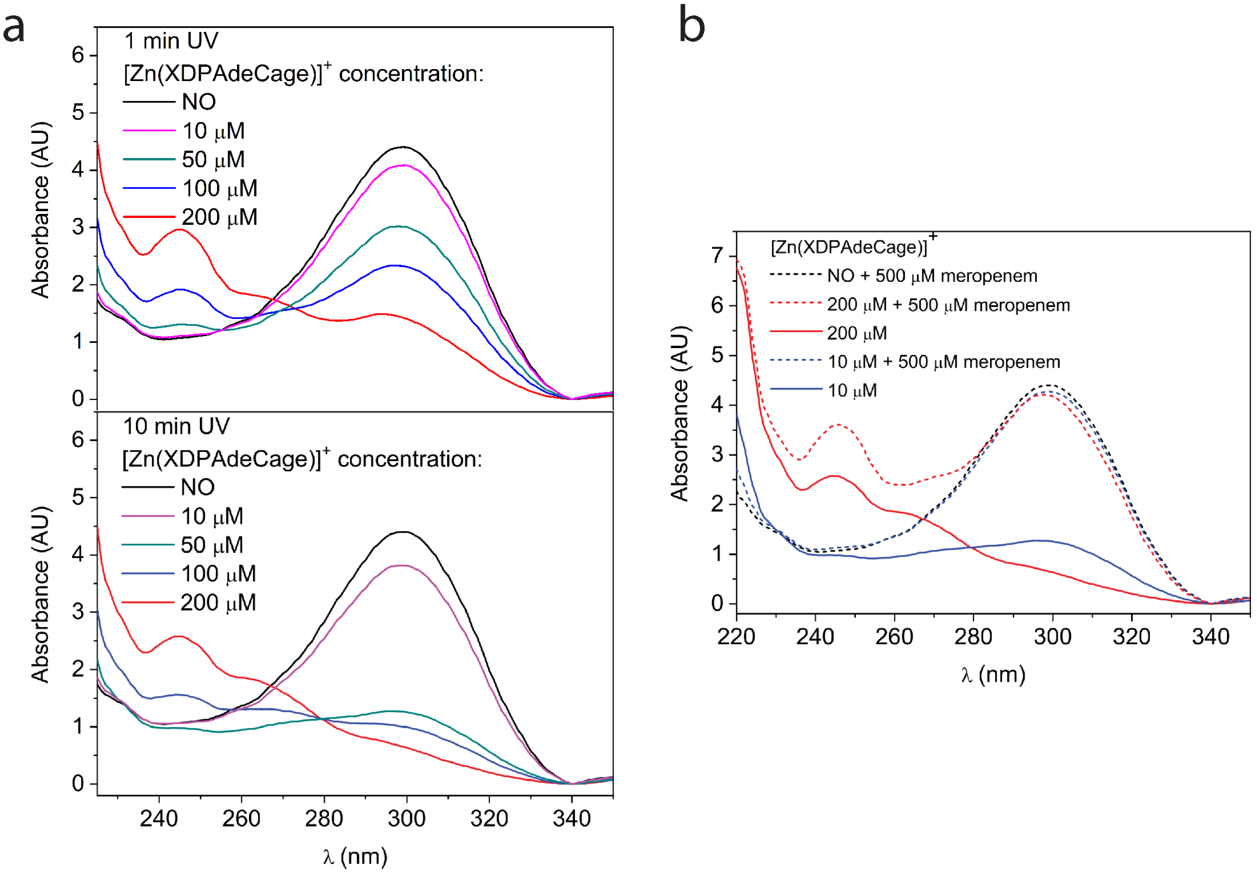

Supplementary Figure 1. Photolysis of $[Zn(XDPAdeCage)]^+$ with 347 nm UV light in solution triggers cleavage of meropenem in a presence of L1 from *S. maltophilia*. **a.** Activity of L1 MBL against meropenem. Prior experiment zinc ions were removed from L1 by dialysis against buffer B containing EDTA. For the measurements we used 500 μM of meropenem with 0.1 μM of L1 MBL. **b.** Control spectra depicting absorption of meropenem and $[Zn(XDPAdeCage)]^+$ without L1 MBL.

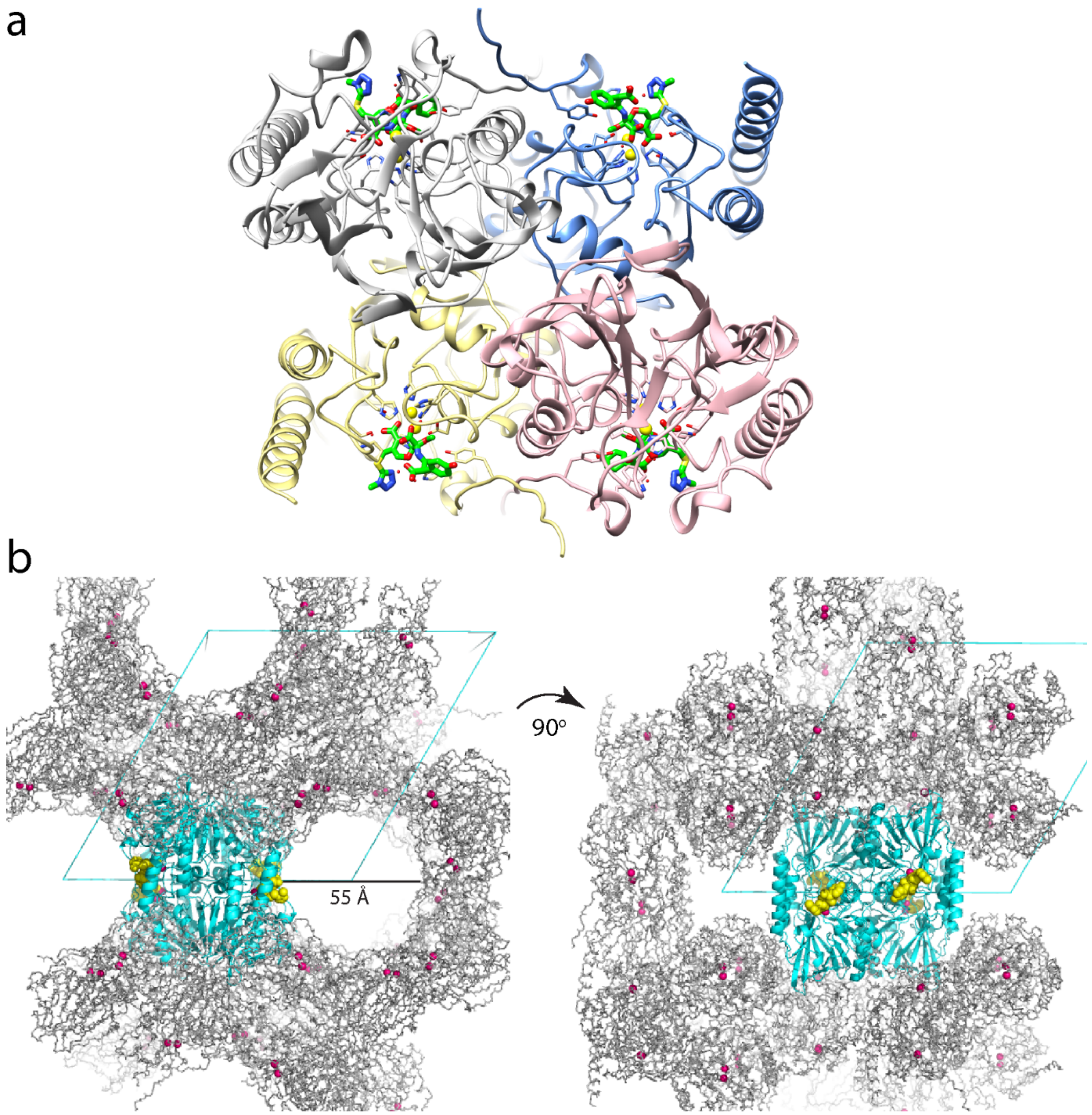

Supplementary Figure 2. Structure of L1 tetramer in complex with hydrolyzed moxalactam. **a.** Cartoon representation of L1 MBL tetramer, subunits are generated by applying crystallographic symmetry. **b.** Crystal packing of L1 MBL at $P6_422$ space group. Symmetry mates of L1 crystal structure were depicted as grey sticks, the secondary structures for tetramer biological assembly were shown as cyan, and the moxalactam bound to L1 tetramer was shown as yellow. To illustrate the localization of all active sites in the crystal of L1 MBL the zinc atoms were illustrated as purple spheres.

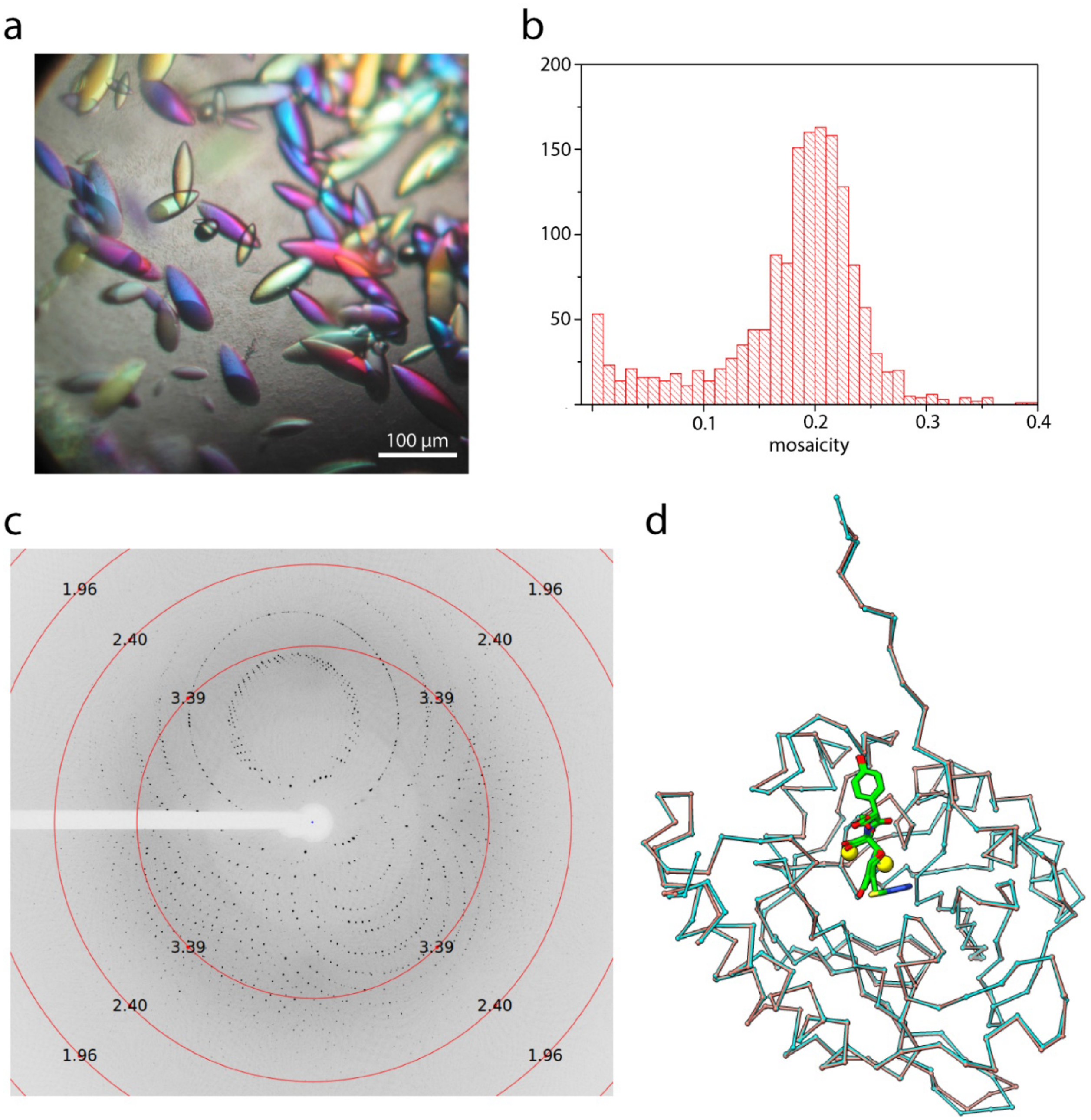

Supplementary Figure 3. Crystals of L1 from *S. maltophilia* grown using batch crystallization. **a.** Crystallization conditions were established and repeated for all collected data sets (more than 20 times). Crystal nucleation was initiated by micro-seeding resulting in high homogeneity of grown crystals. **b.** Histogram of mosaicity values from monochromatic diffraction data obtained during data collection at 19-ID beamline at APS as a part of L1 SSX experiments. **c.** Diffraction image obtained from L1 crystal deposited on nylon mesh using "pink" beam during TR-SSX experiment at 14-ID-B BioCARS beamline at APS. **d.** Superposition of L1 MBL structure from "pink" beam SSX (PDB entry 7L91) and cryo-cooled crystal (PDB entry 6UAC).

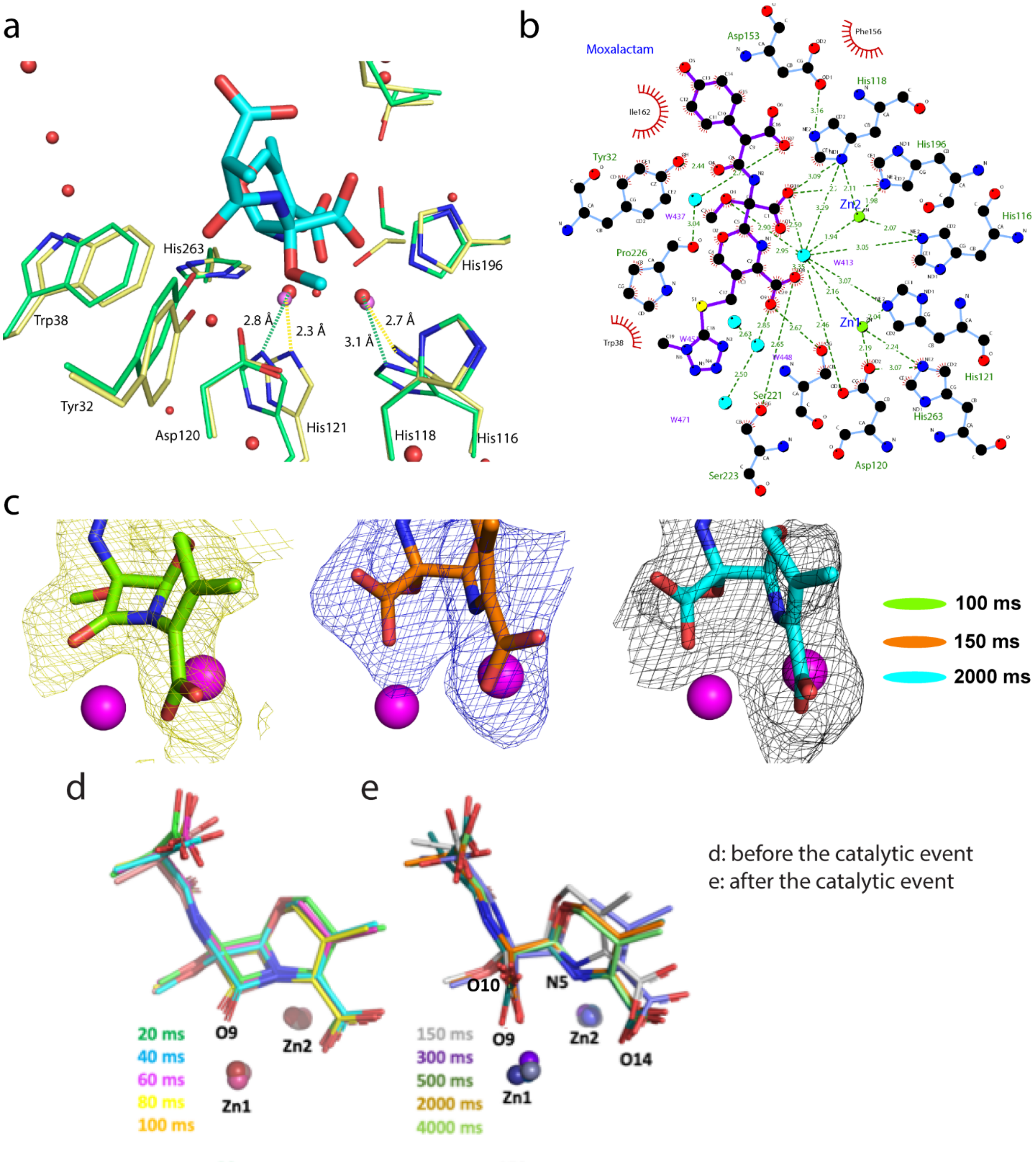

Supplementary Figure 4. Details of active site of L1 MBL. **a.** Comparison of sidechain positions in an active site of L1 MBL: crystal structure after EDTA treatment with waters in active site (green sticks), crystal structure at 2000 ms time point (yellow sticks – sidechain, moxalactam—aqua, zincs purple) **b.** Hydrogen bonds and hydrophobic interactions of the hydrolyzed moxalactam bound by L1 active site, structure determined using "pink" beam SSX (PDB entry 7L91). Moxalactam illustrated as purple stick,

two zinc ions depicted as green spheres. Diagram made using LIGPLOT v.4.5.3 software [1]. **c.** Time-resolved details of electron density observed *in crystallo* reaction of β-lactam ring cleavage of moxalactam by L1 MBL. The 2Fo-Fc polder OMIT maps countered at 2.0 σ level (carved at 2 Å) around moxalactam. Maps depicted as the olive mesh for 100 ms, blue mesh for 150 ms, and black mesh for 2000 ms, zinc ions are in pink. Polder OMIT maps calculated using Phenix [2] with the exclusion of bulk solvent in radius of 5 Å from moxalactam, resolution factor 0.25 were used during calculation. **d.** Comparison of moxalactam movement in the L1 MBL active site observed from 20-100 ms after UV-pulse (before reaction). **e.** Moxalactam conformation changes after reaction, time steps 150-4000 ms.

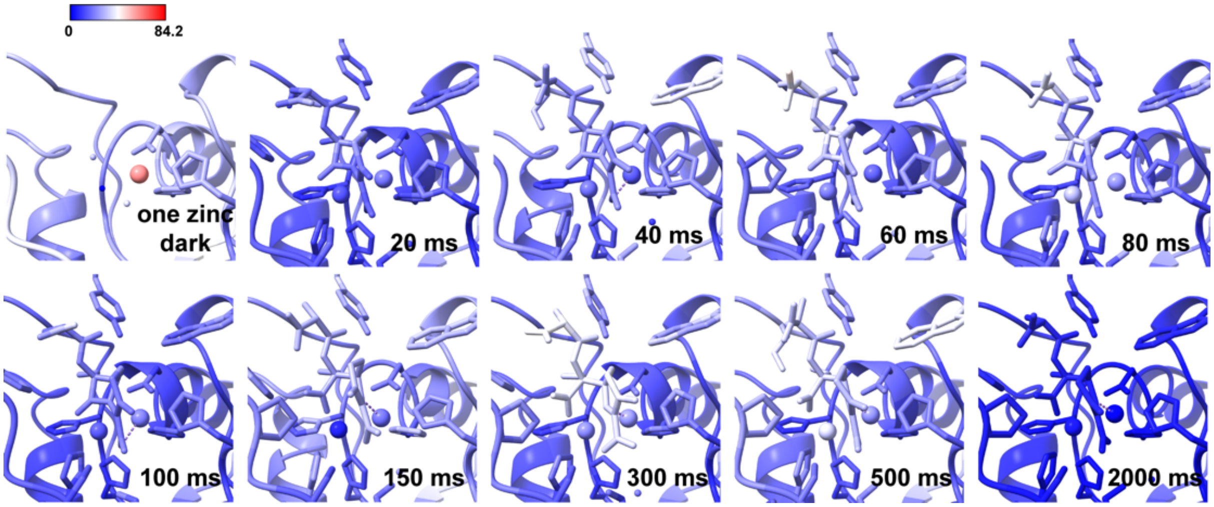

Supplementary Figure 5. B-factors change over the time course of β-lactam ring cleavage of moxalactam by L1 MBL. Crystal structures of L1 MBL dark-set and time-resolved structures determined at time points from 20 to 2000 ms. The bar (from high B-factor red to low B-factor blue) shows the range of B-factors.

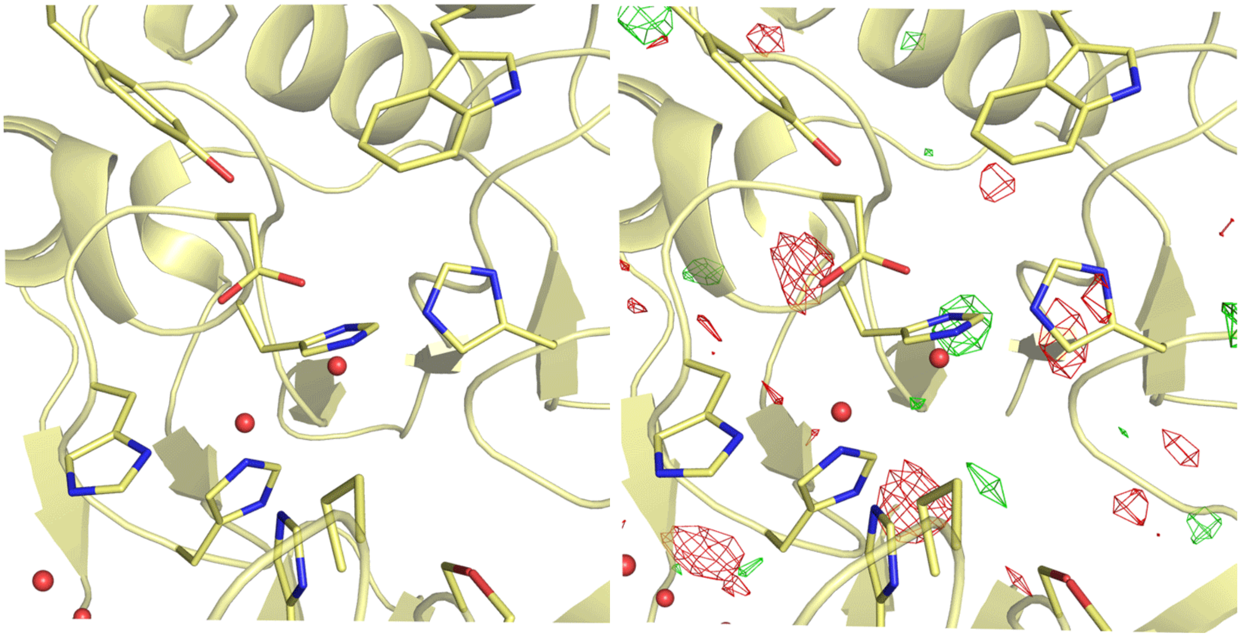

Supplementary Figure 6. Snapshots of moxalactam cleavage by L1 from *S. maltophilia* captured by TR-SSX (20 – 4000 ms). Protein is in yellow, zinc ions in magenta, water molecules are labelled red, moxalactam is in stick representation with carbon atoms in green prior β-lactam cleavage, orange right after cleavage (150 ms) and blue in the final product during conformational adjustments. **a.** shows model of moxalactam in the L1 active site; **b.** shows electron density for the ligand (blue mesh) and additional electron density in the active site (green is positive and red in negative). The 2Fo-Fc map contoured at 1.0 σ level (carved at 1.4 Å). The Fo-Fc electron density maps labeled as green and red, respectively for 3.2 and -3.2 σ level.

Supplementary Table 1A. SSX data collection and processing statistic of L1 β-lactamase crystals.

	**hydrolyzed mox**	**dark**	**no zinc**	**one zinc**	**20 ms**	**40 ms**	**60 ms**
Diffraction source	14-ID-B APS	14-ID-B APS	19-ID APS	19-ID APS	14-ID-B APS	14-ID-B APS	14-ID-B APS
Wavelength (Å)	1.02-1.18	1.02-1.18	0.9792	0.9792	1.02-1.18	1.02-1.18	1.02-1.18
Temperature (K)	295	295	295	295	295	295	295
Detector	RAYONIX MX340-HS	RAYONIX MX340-HS	PILATUS3 X 6M	PILATUS3 X 6M	RAYONIX MX340-HS	RAYONIX MX340-HS	RAYONIX MX340-HS
X-ray pulse length	24 x 100 ps	24 x 100 ps	40 ms	60 ms	24 x 100 ps	24 x 100 ps	24 x 100 ps
Beam size (µm)	60 x 60	60 x 60	50 x 50	75 x 75	60 x 60	60 x 60	60 x 60
APS bunch mode (pulses)	24	24	–	–	24	24	24
X-ray dose (kGy)	16.8	16.8	16.4	11.4	16.8	16.8	16.8
Laser size (µm)	off	off	–	–	100 x 80	100 x 80	100 x 80
ALEX step size (µm)	200 x 200	130 x 180	50 x 50	75 x 75	200 x 250	200 x 250	200 x 250
Scan size x/y (steps)	41 x 55	60 x 60	120 x 150	175 x 220	42 x 45	42 x 45	42 x 45
Detector distance (mm)	300	300	350	350	300	300	300
Number of chips	1	1	1	1	3	2	3
Number of collected images	19,316	5,000	38,500	18,000	5,670	3,780	5,670
Hit-finder (spots/intensity)	60/60	50/60	–	–	30/30	30/30	30/30
Number of crystal hits	7,816	1,210	5191	3374	2,578	2,921	2,324
Crystal hit rate (%)	40.5	24.2	13.5	18.7	45.5	77.3	41.0
Number of indexed images	2301	355	4550	2947	526	533	467
Indexing hit rate (%)	29.4	29.3	87.6	87.3	20.4	18.2	20.1
**Number of merged lattices**	**583**	**143**	**4550**	**2947**	**214**	**198**	**161**
Images merged/indexed (%)	25.3	40.3	100	100	40.7	37.1	34.5
**PDB Accession Code**	**7L91**	**7UHR**	**7UHS**	**7UHT**	**7UHH**	**7UHI**	**7UHJ**

Supplementary Table 1B. SSX data collection and processing statistic of L1 β-lactamase crystals.

	**80 ms**	**100 ms**	**150 ms**	**300 ms**	**500 ms**	**2000 ms**	**4000 ms**
Diffraction source	14-ID-B APS	14-ID-B APS	14-ID-B APS	14-ID-B APS	14-ID-B APS	14-ID-B APS	14-ID-B APS
Wavelength (Å)	1.02-1.18	1.02-1.18	1.02-1.18	1.02-1.18	1.02-1.18	1.02-1.18	1.02-1.18
Temperature (K)	295	295	295	295	295	295	295
Detector	RAYONIX MX340-HS	RAYONIX MX340-HS	RAYONIX MX340-HS	RAYONIX MX340-HS	RAYONIX MX340-HS	RAYONIX MX340-HS	RAYONIX MX340-HS
X-ray pulse length	24 x 100 ps	24 x 100 ps	48 x 100 ps	24 x 100 ps	24 x 100 ps	48 x 100 ps	48 x 100 ps
Beam size (µm)	60 x 60	60 x 60	30 x 30	60 x 60	60 x 60	30 x 30	30 x 30
APS bunch mode (pulses)	24	24	48	48	48	48	48
X-ray dose (kGy)	16.8	16.8	33.6	33.6	33.6	33.6	33.6
Laser size (µm)	100 x 80	100 x 80	100 x 80	100 x 80	100 x 80	100 x 80	100 x 80
ALEX step size (µm)	42 x 45	42 x 45	42 x 36	42 x 35	42 x 30	42 x 30	42 x 24
Scan size x/y (steps)	200 x 250	200 x 250	200 x 315	200 x 315	200 x 380	200 x 380	200 x 450
Detector distance (mm)	300	300	300	300	300	300	300
Number of chips	3	3	4	3	4	7	5
Number of collected images	5,753	5,670	6,048	4,410	5,040	7,713	4,032
Hit-finder (spots/intensity)	30/30	30/30	40/30	30/30	30/30	30/30	30/25
Number of crystal hits	2,127	2,746	2,847	1,915	2,339	4,025	2,200
Crystal hit rate (%)	37.0	48.4	47.1	43.4	46.4	52.2	54.6
Number of indexed images	316	476	734	230	379	543	347
Indexing hit rate (%)	14.9	17.3	25.8	12.0	16.2	13.5	15.8
**Number of merged lattices**	**122**	**191**	**316**	**99**	**154**	**265**	**166**
Images merged/indexed (%)	38.6	40.1	43.1	43.0	40.6	48.8	47.8
**PDB Accession Code**	**7UHK**	**7UHL**	**7UHM**	**7UHN**	**7UHO**	**7UHP**	**7UHQ**

Supplementary Table 2A. L1 β-lactamase crystallographic data and refinement statistics.

	hydrolyzed mox	dark	no zinc	one zinc	20 ms	40 ms	60 ms
**Ligands**	two zincs hydrolyzed mox	one zinc in active site	two waters in active site	one zinc	two zincs substrate mox	two zincs substrate mox	two zincs substrate mox
Space group	$P6_422$	$P6_422$	$P6_422$	$P6_422$	$P6_422$	$P6_422$	$P6_422$
a, b, c (Å)	105.85, 105.85, 99.10	105.85, 105.85, 99.10	105.95, 105.95, 99.89	106.09, 106.09, 99.77	105.85, 105.85, 99.10	105.85, 105.85, 99.10	105.85, 105.85, 99.10
Resolution range (Å) [a]	33.03 – 2.20	26.01 – 2.20	46.80 – 2.20	46.84 – 2.20	33.03 – 2.20	33.03 – 2.20	28.39 – 2.20
Res. range in upper bin (Å) [a]	(2.30 – 2.20)	(2.30 – 2.20)	(2.24 – 2.20)	(2.24 – 2.20)	(2.30 – 2.20)	(2.30 – 2.20)	(2.30 – 2.20)
No. of unique reflections	14,754 (1,143)	12,094 (593)	17,385 (865)	17,389 (861)	12,926 (729)	12,800 (703)	12,501 (671)
Completeness (%)	86.4 (55.6)	70.8 (28.8)	100.0 (100.0)	100.0 (100.0)	75.7 (35.4)	74.9 (34.11)	73.3 (32.7)
Data redundancy	35.4	9.2	76.5	54.3	13.1	12.1	10.4
R_{merge} (%) [b] or R_{split} (%) [c]	13.4 (12.2)	12.1 (10.4)	24.54 (97.48)	27.73 (82.19)	13.3 (11.9)	13.6 (12.2)	13.1 (11.8)
$CC_{1/2}$ [d]	–	–	95.46 (40.89)	90.84 (52.35)	–	–	–
$\langle I/\sigma(I)\rangle$ or $\langle F^2/\sigma(F^2)\rangle$	30.51	20.2	2.51 (0.39)	2.99 (0.50)	21.1	20.0	19.3
Wilson *B* factor	22.2	13.5	31.7	34.3	11.2	9.2	14.5
**Refinement** (MR model)	7L52	7L52	7L52	7L52	7L91	7L91	7L91
Resolution range (Å)	33.03 – 2.20 (2.34 – 2.20)	26.01 – 2.20 (2.42 – 2.20)	46.80 – 2.20 (2.34 – 2.20)	46.87 – 2.20 (2.34 – 2.20)	33.03 – 2.20 (2.42 – 2.20)	33.03 – 2.20 (2.42 – 2.20)	28.39 – 2.20 (2.42 – 2.20)
Completeness (%)	85.7 (58.5)	70.1 (39.0)	99.4 (97.0)	100.0 (100.0)	75.1 (45.0)	74.5 (44.0)	72.7 (42.0)
No. of unique reflections	14,723 (1,546)	12,031 (1,540)	17,255 (2,589)	17,383 (2,683)	12,887 (1,793)	12,761 (1,750)	12,453 (1,674)
R_{work}/R_{free} [e] (%)	18.14/20.75 (20.30/ 22.16)	22.18/24.36 (27.63/27.23)	22.14/25.77 (32.35/34.12)	21.45/25.39 (32.70/35.34)	22.34/26.20 (30.82/37.84)	23.06/26.61 (28.23/32.32)	20.27/23.83 (24.85/27.90)
Protein chains/atoms	1/1,986	1/1,986	1/1,981	1/1,986	1/1,986	1/1,986	1/1,986
Ligands/Solvent atoms	39/71	1/72	0/35	1/45	25/50	25/53	25/55
Mean B factor ($Å^2$)	26.9	28.9	45.1	37.2	20.6	13.6	18.8
R.m.s.d. bonds (Å)	0.003	0.001	0.001	0.002	0.003	0.002	0.001
R.m.s.d. angles (º)	0.627	0.401	0.430	0.530	0.589	0.519	0.523
**Ramachandran plot** [f]							
Favored (%)	96.2	94.6	95.4	96.6	93.9	95.4	95.0
Allowed (%)	3.8	5.0	4.6	3.4	6.1	4.2	5.0
Outside allowed (%)	0.0	0.4	0.0	0.0	0.0	0.4	0.0
Clashscore	1.0	2.0	2.0	1.3	1.8	2.3	1.0
**PDB Accession Code**	**7L91**	**7UHR**	**7UHS**	**7UHT**	**7UHH**	**7UHI**	**7UHJ**

Supplementary Table 2B. L1 β-lactamase crystal crystallographic data and refinement statistics.

	80 ms	100 ms	150 ms	300 ms	500 ms	2000 ms	4000 ms
**Ligands**	two zincs substrate mox	two zincs substrate mox	two zincs hydrolyzed mox	two zincs hydrolyzed mox	two zincs hydrolyzed mox	two zincs hydrolyzed mox	two zincs hydrolyzed mox
Space group	$P6_422$	$P6_422$	$P6_422$	$P6_422$	$P6_422$	$P6_422$	$P6_422$
a, b, c (Å)	105.85, 105.85, 99.10	105.85, 105.85, 99.10	105.85, 105.85, 99.10	105.85, 105.85, 99.10	105.85, 105.85, 99.10	105.85, 105.85, 99.10	105.85, 105.85, 99.10
Resolution range (Å) [a]	31.08 – 2.20	31.08 – 2.20	34.64 – 2.20	33.03 – 2.20	31.08 – 2.20	33.65 – 2.20	33.65-2.20
Res. range in upper bin (Å) [a]	(2.30 – 2.20)	(2.30 – 2.20)	(2.30 – 2.20)	(2.30 – 2.20)	(2.30 – 2.20)	(2.30 – 2.20)	(2.30 – 2.20)
No. of unique reflections	11,936 (595)	12,754 (757)	12,098 (585)	10,982 (452)	12,324 (670)	11,732 (448)	11,599 (499)
Completeness (%)	70.0 (28.9)	74.7 (36.9)	71.0 (28.3)	64.2 (21.9)	72.2 (32.6)	68.6 (21.7)	67.8 (24.3)
Data redundancy	8.0	11.8	15.3	6.5	9.7	12.8	10.1
R_{merge} (%) [b]	12.7 (11.0)	13.4 (11.2)	13.6 (10.3)	12.6 (9.1)	13.3 (11.6)	16.1 (9.3)	12.6 (10.7)
$CC_{1/2}$ [d]	–	–	–	–	–	–	–
$\langle I/\sigma(I)\rangle$ or $\langle F^2/\sigma(F^2)\rangle$	18.3	19.9	23.0	17.3	18.6	18.1	20.5
Wilson *B* factor	11.3	10.1	5.7	9.6	10.1	4.5	7.0
**Refinement** (MR model)	7L91	7L91	7L91	7L91	7L91	7L91	7L91
Resolution range (Å)	31.08 – 2.20 (2.42 – 2.20)	31.08 – 2.20 (2.42 – 2.20)	34.64 – 2.70 (3.09 – 2.70)	33.03 – 2.20 (2.42 – 2.20)	31.08 – 2.20 (2.42 – 2.20)	33.65 – 2.60 (2.98 – 2.60)	33.65-2.20 (2.43-2.20)
Completeness (%)	69.4 (37.0)	74.0 (45.0)	88.6 (79.0)	63.4 (29.0)	71.7 (42.0)	85.2 (73.0)	67.6 (34.0)
No. of unique reflections	11,882 (1,484)	12,691 (1,795)	8,383 (2,314)	10,918 (518)	12,282 (1,645)	8,995 (2,359)	11,547 (1,335)
R_{work}/R_{free}^{e} (%)	19.44/24.38 (24.12/26.11)	20.16/24.53 (26.16/31.36)	23.50/29.01 (33.99/38.45)	19.91/24.63 (23.65/25.47)	20.86/25.24 (24.38/27.54)	25.06/27.54 (32.93/40.44)	23.31/26.44 (29.77/33.58)
Protein chains/atoms	1/1,986	1/1,986	1/1,986	1/1,986	1/1,986	1/1,986	1/1,986
Ligands/Solvent atoms	25/73	25/84	26/45	26/86	26/86	26/47	26/60
Mean B factor ($Å^2$)	16.2	15.3	22.3	15.2	15.0	4.8	24.9
R.m.s.d. bonds (Å)	0.002	0.002	0.002	0.002	0.002	0.002	0.002
R.m.s.d. angles (º)	0.519	0.526	0.543	0.501	0.467	0.505	0.482
**Ramachandran plot** [f]							
Favored (%)	94.6	95.0	91.6	95.8	95.4	94.3	94.3
Allowed (%)	5.0	5.0	7.3	4.2	4.6	5.7	5.3
Outside allowed (%)	0.4	0.0	1.1	0.0	0.0	0.0	0.4
Clashscore	1.8	2.3	3.8	2.0	2.5	3.5	2.5
**PDB Accession Code**	**7UHK**	**7UHL**	**7UHM**	**7UHN**	**7UHO**	**7UHP**	**7UHQ**

[a]Values in parentheses correspond to the highest resolution shell.
[b]$R_{merge} = \Sigma_h\Sigma_j|I_{hj}–<I_h>|/\Sigma_h\Sigma_jI_{hj}$, where I_{hj} is the intensity of observation j of reflection h.
[c]R_{split} as defined by White [3].
[d]$CC_{1/2}$ as defined by Karplus and Diederichs [4]
[e]$R = \Sigma_h|Fo|–|Fc|/\Sigma_h|Fo|$ for all reflections, where Fo and Fc are observed and calculated structure factors, respectively. R_{free} is calculated analogously for the test reflections, randomly selected and excluded from the refinement.
[f]As defined by Molprobity [5].

Supplementary Table 3. Atomic distances (Å) after comparison of atom positions at specific time point to 20 ms data. (Module from difference of atomic distances measured at T and at T_{20ms})

	**40 ms**	**60 ms**	**100 ms**	**150 ms**	**300 ms**	**500 ms**	**2000 ms**	**4000 ms**
**Zn1 - O9**	0.23	0.14	0.24	0.28	0.49	**0.63**	**0.67**	**0.63**
**Zn1 - C8**	0.32	0.04	0.29	0.45	0.19	0.12	0.04	0.14
**Zn1 - C6**	0.40	0.03	0.36	0.08	**0.51**	0.06	0.43	0.08
**Zn1 - C7**	0.39	0.02	0.30	0.04	0.34	0.04	0.44	0.15
**Zn1 - N5**	0.27	0.03	0.26	**0.59**	0.04	0.05	0.21	0.13
**Zn1 - C4**	0.07	0.08	0.13	**1.04**	**0.51**	0.17	0.02	0.21
**Zn2 - O9**	0.02	0.04	0.09	**0.60**	0.28	0.39	0.39	0.49
**Zn2 - C8**	0.13	0.08	0.15	**0.59**	**0.69**	**0.57**	**0.73**	**0.65**
**Zn2 - C6**	0.39	0.17	0.31	0.11	0.45	0.08	0.10	0.11
**Zn2 - C7**	0.31	0.18	0.17	0.06	0.21	0.10	0.13	0.16
**Zn2 - N5**	0.09	0.02	0.21	0.36	0.46	**0.51**	**0.52**	**0.54**
**Zn2 - C4**	0.23	0.29	0.13	0.43	0.16	0.08	0.16	0.14
**N5 - C8**	0.00	0.00	0.00	**1.24**	**1.23**	**1.21**	**1.35**	**1.32**
**N5 - O9**	0.00	0.00	0.00	0.37	0.43	**0.54**	0.41	0.40
**N5 - C4**	0.00	0.00	0.00	0.01	0.01	0.01	0.01	0.01
**N5 - O14**	0.01	0.00	0.00	**0.91**	**0.87**	**0.92**	**0.94**	**0.93**
**N5 - C2**	0.01	0.00	0.00	0.24	0.15	0.18	0.19	0.19
**D120 OD1 - N5**	0.20	0.26	0.02	0.02	0.29	0.07	0.11	0.06
**D120 OD2 - N5**	0.31	0.05	0.07	0.09	0.29	0.38	0.47	0.39
**D120 OD1 - Zn2**	0.27	0.19	0.21	0.10	0.13	0.14	0.33	0.10

Supplementary Table 4. Atomic distances (Å) measured during TR reaction of catalysis of the β-lactam ring by L1 MBL.

	20 ms	40 ms	60 ms	100 ms	150 ms	300 ms	500 ms	2000 ms	4000 ms
**Zn1 – O9**	2.76	2.53	2.63	2.53	2.49	2.27	2.14	2.10	2.13
**Zn1 – C8**	3.20	2.88	3.16	2.91	3.65	3.39	3.32	3.24	3.35
**Zn1 – C6**	4.66	4.25	4.69	4.30	4.73	4.14	4.60	4.22	4.58
**Zn1 – C7**	4.28	3.89	4.30	3.98	4.31	3.94	4.24	3.84	4.12
**Zn1 – N5**	3.70	3.43	3.72	3.44	4.28	3.66	3.74	3.49	3.83
**Zn1 – C4**	4.25	4.18	4.33	4.12	5.29	4.76	4.42	4.23	4.47
**Zn2 – O9**	4.11	4.09	4.07	4.02	3.51	3.84	3.72	3.73	3.63
**Zn2 – C8**	3.52	3.40	3.44	3.38	4.12	4.21	4.10	4.26	4.17
**Zn2 – C6**	3.34	2.95	3.17	3.03	3.23	2.89	3.26	3.24	3.23
**Zn2 – C7**	4.12	3.81	3.94	3.95	4.06	3.91	4.02	3.99	3.96
**Zn2 – N5**	2.76	2.67	2.78	2.55	2.40	2.30	2.25	2.24	2.21
**Zn2 – C4**	2.88	3.10	3.17	2.75	3.31	3.04	2.96	3.04	3.02
**N5 – C8**	1.37	1.37	1.37	1.37	2.61	2.60	2.58	2.72	2.70
**N5 – O9**	2.36	2.36	2.36	2.36	2.73	2.79	2.90	2.77	2.76
**N5 – C4**	1.38	1.39	1.38	1.38	1.37	1.38	1.37	1.38	1.37
**N5 – O14**	3.61	3.62	3.62	3.61	2.70	2.74	2.70	2.67	2.68
**N5 – C2**	2.53	2.53	2.53	2.53	2.76	2.68	2.71	2.72	2.72
**D120 OD1 – N5**	4.56	4.76	4.81	4.54	4.57	4.84	4.49	4.44	4.50
**D120 OD2 – N5**	3.89	4.20	3.94	3.83	3.80	3.60	3.51	3.42	3.50
**D120 OD1 – Zn2**	3.37	3.10	3.18	3.58	3.46	3.49	3.22	3.04	3.26

Supplementary Table 5. Occupancies and B-factors of moxalactam and zincs atoms in L1 β-lactamase crystal structures.

	**hydrolyzed mox**	**dark**	**no zinc**	**one zinc**	**20 ms**	**40 ms**	**60 ms**
**Ligands**	two zincs hydrolyzed mox	one zinc in active site	two waters in active site	one zinc	two zincs substrate mox	two zincs substrate mox	two zincs substrate mox
Mean B factor (Å^2)	26.9	28.9	45.1	37.2	20.6	13.6	18.8
Occupancy moxalactam (%)	100	-	-	-	70	80	83
B-factor (Å^2) moxalactam	41.0	-	-	-	21.2	13.2	21.3
Occupancy Zn1 (%)	82.0	-	-	79.0	59.0	49.0	53.0
B-factor (Å^2) Zn1	14.4	-	-	29.1	17.6	12.8	19.1
Occupancy Zn2 (%)	80.0	100	-	-	58	77	73
B-factor (Å^2) Zn2	13.0	62.0	-	-	14.4	18.2	15.2
**PDB Accession Code**	**7L91**	**7UHR**	**7UHS**	**7UHT**	**7UHH**	**7UHI**	**7UHJ**
	**80 ms**	**100 ms**	**150 ms**	**300 ms**	**500 ms**	**2000 ms**	**4000 ms**
**Ligands**	two zincs substrate mox	two zincs substrate mox	two zincs hydrolyzed mox	two zincs hydrolyzed mox	two zincs hydrolyzed mox	two zincs hydrolyzed mox	two zincs hydrolyzed mox
Mean B factor (Å^2)	16.2	15.3	22.3	15.2	15.0	4.8	24.9
Occupancy moxalactam (%)	87	73	77	68	81	90	85
B-factor (Å^2) moxalactam	16.2	15.2	22.3	29.3	15.2	5.8	28.0
Occupancy Zn1 (%)	66	48	48	60	49	88	61
B-factor (Å^2) Zn1	28.5	13.9	5.9	21.5	18.6	4.2	27.9
Occupancy Zn2 (%)	85	73	86	52	78	100	66
B-factor (Å^2) Zn2	22.2	19.5	17.8	23.4	21.7	1.3	14.2
**PDB Accession Code**	**7UHK**	**7UHL**	**7UHM**	**7UHN**	**7UHO**	**7UHP**	**7UHQ**

Supplementary Table 6. Values of B-factors for specific atoms in moxalactam observed in L1 MBL during the time-resolved reaction. Crystal structures of L1 were determined by pink-beam serial crystallography. Numbers used for the description of atom position in moxalactam were depicted in Figure 3a.

	20 ms	40 ms	60 ms	80 ms	100 ms	150 ms	300 ms	500 ms	2000 ms	4000 ms	hydrolyzed mox.
**C2**	21.66	22.67	26.41	26.25	18.13	28.17	37.54	20.05	5.91	29.16	39.38
**C3**	21.28	18.43	24.73	20.68	18.67	28.00	34.88	22.01	6.38	28.81	42.00
**C4**	21.41	16.75	25.71	21.96	17.96	27.36	31.70	17.35	5.64	28.37	37.91
**C6**	21.72	17.76	28.32	24.12	21.14	25.94	32.06	21.38	5.18	28.76	35.14
**C7**	22.33	21.19	32.63	28.36	20.36	25.14	34.63	23.63	5.41	28.55	33.24
**C8**	21.63	17.92	25.65	22.79	19.20	23.38	34.53	22.74	5.23	28.69	28.49
**C13**	21.41	14.50	25.40	21.56	18.71	28.11	31.22	20.78	4.07	28.23	36.67
**N5**	21.55	15.10	29.72	23.11	19.00	26.13	31.78	17.79	4.90	28.16	35.76
**N17**	22.10	17.03	27.49	22.39	19.51	26.91	34.63	20.06	6.06	28.62	35.65
**O1**	22.03	25.54	29.53	25.12	23.20	27.37	34.91	21.47	5.51	29.30	37.53
**O9**	21.26	18.25	23.70	21.73	17.62	22.63	32.62	21.00	4.25	28.55	23.86
**O10**	-	-	-	-	-	22.72	36.83	21.55	5.24	28.91	26.67
**O11**	22.04	17.83	27.73	27.63	22.86	24.34	30.70	19.65	5.03	28.13	32.30

Supplementary References

1. Laskowski, R. A. & Swindells, M. B. LigPlot+: Multiple ligand-protein interaction diagrams for drug discovery. *J. Chem. Inf. Model.* **51**, 2778–2786 (2011).

2. Liebschner, D. *et al.* Polder maps: improving OMIT maps by excluding bulk solvent. *Acta Crystallogr. Sect. D, Struct. Biol.* **73**, 148–157 (2017).

3. White, T. A. *et al.* Crystallographic data processing for free-electron laser sources. *Acta Crystallogr. Sect. D Biol. Crystallogr.* **69**, 1231–1240 (2013).

4. Karplus, P. A. & Diederichs, K. Linking crystallographic model and data quality. *Science* **336**, 1030–1033 (2012).

5. Davis, I. W., Murray, L. W., Richardson, J. S. & Richardson, D. C. MOLPROBITY: structure validation and all-atom contact analysis for nucleic acids and their complexes. *Nucleic Acids Res.* **32**, 615-619 (2004).
