# Supplementary material for: Time-resolved β-lactam cleavage by L1 metallo-β-lactamase
Source: Nat Commun. 2022 Nov 30;13:7379. doi: 10.1038/s41467-022-35029-3 (PMC9712583; doi:10.1038/s41467-022-35029-3)
Supplement: Supplementary file 3 — Description of Additional Supplementary files [file 41467_2022_35029_MOESM3_ESM.pdf]

Description of Additional Supplementray files

File name: Supplementary Movie 1

Description: Snapshots of moxalactam cleavage by L1 from *S. maltophilia* captured by TR-SSX (20 – 4000 ms). Protein is in yellow, zinc ions in magenta, water molecules are labelled red, moxalactam is in stick representation with carbon atoms in green prior β-lactam cleavage, orange right after cleavage (150 ms) and blue in the final product during conformational adjustments. Movie shows conformational changes to model of moxalactam in the L1 active site.

File name: Supplementary Movie 2

Description: Snapshots of moxalactam cleavage by L1 from *S. maltophilia* captured by TR-SSX (20 – 4000 ms). Protein is in yellow, zinc ions in magenta, water molecules are labelled red, moxalactam is in stick representation with carbon atoms in green prior β-lactam cleavage, orange right after cleavage (150 ms) and blue in the final product during conformational adjustments. Movie shows electron density for the ligand (blue mesh) and additional electron density in the active site (green is positive and red in negative). The 2Fo-Fc map contoured at 1.0 σ level (carved at 1.4 Å). The Fo-Fc electron density maps labeled as green and red, respectively for 3.2 and -3.2 σ level.
