# Supplementary figures and images for: Time-resolved β-lactam cleavage by L1 metallo-β-lactamase
Source: Nat Commun. 2022 Nov 30;13:7379. doi: 10.1038/s41467-022-35029-3 (PMC9712583; doi:10.1038/s41467-022-35029-3)

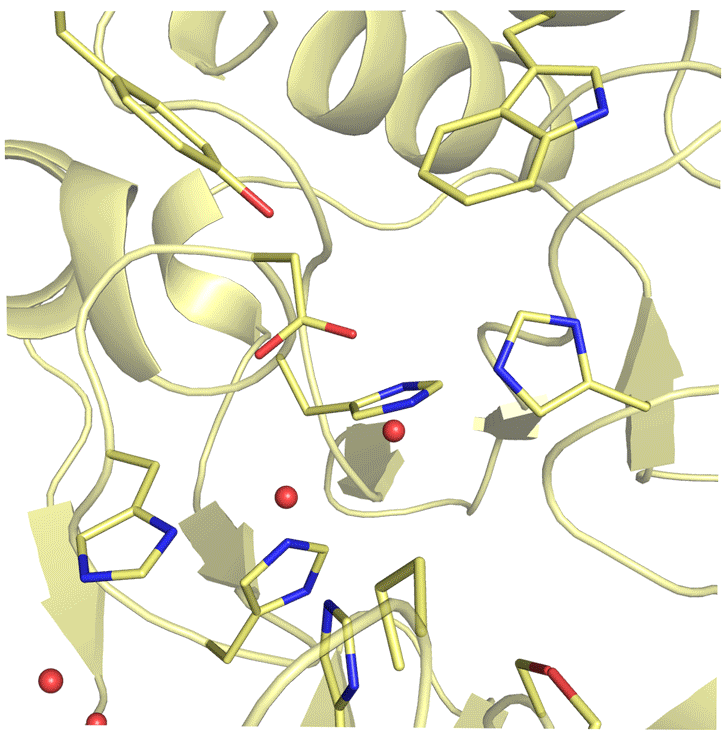

Supplement: Supplementary file 4 — Supplementary Movie 1 [file 41467_2022_35029_MOESM4_ESM.gif]

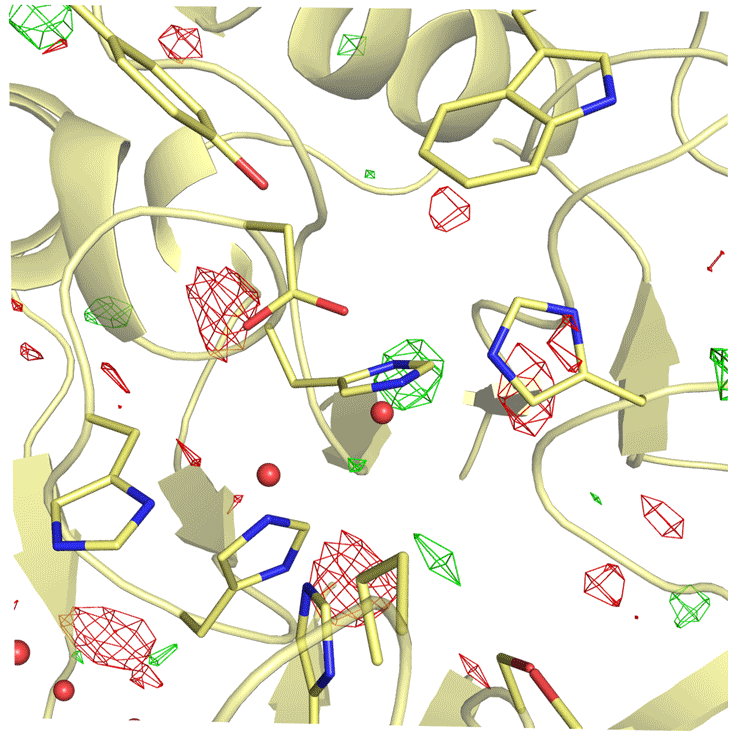

Supplement: Supplementary file 5 — Supplementary Movie 2 [file 41467_2022_35029_MOESM5_ESM.gif]
